# Multiphase abdomen-pelvis CT in women of childbearing potential (WOCBP): Justification and radiation dose

**DOI:** 10.1097/MD.0000000000018485

**Published:** 2020-01-24

**Authors:** Huda Al Naomi, Antar Aly, Mohamad Hassan Kharita, Shatha Al Hilli, Amal Al Obadli, Ramandeep Singh, Madan M. Rehani, Mannudeep K. Kalra

**Affiliations:** a Hamad Medical Corporation, Doha, Qatar; b Massachusetts General Hospital and Harvard Medical School, Boston MA.

**Keywords:** CT, justification, multiphase CT, radiation dose, women of childbearing potential

## Abstract

To assess justification and radiation doses of abdomen-pelvis CT in women of childbearing potential (WOCBP) scanned in 2 tertiary hospitals in Qatar.

The local ethical committee approved retrospective study of 451 WOCBP (14–55 years) who underwent abdomen-pelvis CT examinations. Patients’ age, clinical indications for ordered CT, scanner types and vendors, number and type of scan phases (non-contrast, arterial, portal venous, and/or delayed phases), and radiation dose descriptors (CT dose index volume - CTDIvol and dose length product- DLP) were recorded. Patients undergoing simultaneous chest-abdomen-pelvis CT were excluded. We classified the clinical indications for all 451 CT into indicated and unindicated based on the ACR Appropriateness Criteria. Information regarding the date of last menstrual period, likelihood of pregnancy, and if available, results of the pregnancy test were recorded. Data were analyzed with descriptive statistics (median and inter-quartile range) and analysis of variance (ANOVA).

None of the patients were pregnant at the time of their scanning. Amongst the 673 phases acquired for multiphase abdomen-pelvis CT in 451 patients, the 47% unindicated phases (315/673) included non-contrast (122/673, 18%), arterial (33/673, 5%), portal venous (125/673, 19%) and delayed (35/673, 5%) phases. The respective median DLP for indicated and unindicated phases were 266 and 758 mGy.cm (*P* < .0001).

Multiphase abdomen-pelvis CT exams are frequent but seldom justified in WOCBP. They lead to a substantial increase in unindicated radiation dose compared to a single-phase CT.

## 1 Introduction

Use of CT in modern medicine has increased worldwide. In 2018, about 88 million CT examinations were performed in the United States alone.^[1]^ Concerns over its usage have raised awareness and led to calls for reducing radiation dose associated with CT scanning. These have led to the introduction of various CT technologies including automatic tube current modulation and tube potential selection techniques, efficient detector configuration, and iterative reconstruction techniques to enable dose reduction while maintaining diagnostic confidence.^[2–4]^ Numerous studies have reported radiation dose reduction with these technologies and other innovative methods with clinical indication-based CT protocols, reduction in applied scan factors (tube current, tube potential) and scan length.^[2–4]^ A few studies have evaluated the need for decreasing scan phases to reduce associated radiation doses with multiphase CT examinations of the abdomen-pelvis.^[5–7]^

Abdomen-pelvis region is amongst the most imaged body region with CT; abdomen-pelvis CT exams represent close to one-third of all CT examinations.^[1]^ Due to low contrast organs and abnormalities in the liver, kidneys, pancreas, and spleen, abdomen-pelvis CT exams are often performed at higher dose levels compared to the chest. Since abnormalities demonstrate enhancement in different phases of contrast circulation in the abdomen-pelvis, multiphase CT exams are frequent with the acquisition in non-contrast, arterial, portal venous, and delayed phases of contrast enhancement. Multiphase abdomen-pelvis CT exams result in a substantial increase in radiation dose compared to single-phase CT exams. Although unjustified multiphase CT exams must be avoided, it is important to reduce its frequency amongst children and in women of childbearing potential. The latter group of patients in the conservative Middle-East countries poses special challenges since the acquisition of written informed consent about the likelihood of pregnancy is constrained by the prevailing societal etiquettes and norms. Under such circumstances, justification and frequency of multiphase CT warrant attention. We assessed justification and radiation doses of abdomen-pelvis CT in women of childbearing potential scanned in two tertiary hospitals in Qatar.

## 2 Materials and methods

### 2.1 Approval and financial disclosure

The local ethical committee approved the observational, retrospective study. A research grant from the Qatar National Research Fund (QNRF) supported the study. A study co-author (MKK) has received research grants from Siemens Healthineers and Riverain Inc. for unrelated projects. There are no other financial disclosures pertaining to this study. All authors had complete access to the study data and the manuscript.

### 2.2 Patients

Our study included consecutive 451 women of childbearing potential between the ages of 14 to 55 years who underwent abdomen-pelvis CT examinations between 2015 and 2018. The patients underwent scanning in one of the 2 hospitals belonging to the Hamad Medical Corporation, Qatar, Doha, in Hamad General Hospital, Doha, Qatar (n = 395/451 patients) and Al Wakra Hospital, Wakra, Qatar (n = 56/451 patients). A certified CT technologist identified these patients from the common PACS and the radiology information system (RIS). Patients who underwent combined chest-abdomen-pelvis CT, dedicated liver, renal mass, or adrenal protocol CT, or CT angiography were excluded.

For all patients, we recorded their age, and the date of the last menstrual period (LMP) at the time of their CT exams. Per the hospital policy in Qatar, since a single woman of childbearing potential (either unmarried, divorced, or widowed) are not queried about the likelihood of pregnancy, and were not asked to take a pregnancy test prior to any procedure (including CT scanning), a written informed consent is obtained from such women prior to their radiological procedure including CT. Only the married women of childbearing potential who were uncertain of their LMP or had missed their LMP, had a urine pregnancy test prior to their CT exams as per the hospital policy. When performed, results of the pregnancy tests were recorded from the RIS. For the women who signed the informed consent prior to their CT, we reviewed the electronic medical records to determine if they were pregnant at the time of CT from their follow-up clinical records.

### 2.3 Scanners and scan protocols

All patients underwent scanning on one of the 4 multidetector-row CT scanners (64-row CT, Siemens Sensation 64; 256-row CT, Siemens SOMATOM Flash, 128-row CT, Siemens Definition Edge, Siemens Healthineers, Forchheim, Germany) in Hamad General Hospital, or on a single 256-row multidetector CT (Phillips Brilliance iCT, Phillips Healthcare, Eindhoven, The Netherlands) in Al Wakra Hospital.

The department of radiology at Hamad General Hospital developed and implemented all scan protocols and phases at both the participating institutions. Regardless of the scanner and the location, all included CT exams were performed with automatic tube current modulation (Care Dose 4D, Siemens, or Z-AEC, Phillips), 120 kV, 0.5 to 0.8 second gantry rotation time, and a pitch of 0.9–1.2:1. All abdomen-pelvis CT exams (non-contrast, arterial and portal venous phases) extended from the top of the liver to the pubic symphysis. Most delayed images were acquired through the liver only.

The number and type (non-contrast, arterial, portal venous, or delayed phase) of scan phases for each CT examination were recorded. We recorded radiation dose descriptors, volume CT dose index (CTDIvol) and dose length product (DLP), from the dose information page for each scan phase. Total DLP (sum of DLP of all scan phases) was also recorded. As per recommendation, CTDIvol > 5 mGy were rounded to the nearest whole number; CTDIvol < 5 mGy were rounded to the nearest single decimal point.^[8]^

### 2.4 Justification of CT examinations and scan phases

Clinical indications for each CT were obtained from the shared RIS between the 2 hospitals. We reviewed clinical indications for all 451 CT examinations to determine their justification based on the American College of Radiology (ACR) Appropriateness Criteria.^[9]^ The ACR Appropriateness Criteria classifies the imaging tests for different clinical indications into usually not appropriate, may be appropriate, and usually appropriate. We classified each CT exam as unjustified (if they are deemed usually not appropriate per the ACR Criterion) or justified (if they are either appropriate or usually appropriate).

For multiphase abdomen-pelvis CT examinations, appropriateness for each phase was assessed based on the recommendations within the ACR Appropriateness Criteria. Phases not recommended for acquisition in these guidelines for specified clinical indications were labeled as unjustified, while recommended phases were called justified. For patients who underwent multiphase abdomen-pelvis CT, the average number of unjustified phases per patient was estimated. In addition, we determined the most frequent unjustified phases for these examinations.

### 2.5 Statistical analyses

Data were compiled and analyzed in Microsoft Excel (Microsoft Inc., Redmond, Washington). Descriptive analyses were performed to obtain median and inter-quartile range for CTDIvol and DLP for each scan phase. Scanner-specific stratification of radiation doses was not performed due to the small sample size and to avoid over-testing.

Separately for 1, 2, 3, and 4-phase CT exams, Student's *t* test and one-way analysis of variance (ANOVA) were performed to determine differences in CTDIvol and DLP between different scan phases. Weights of patients who underwent single vs multiphase abdomen-pelvis CT were also compared. A *P* value of less than .05 was considered statistically significant.

## 3 Results

### 3.1 Informed consent

Amongst the 451 patients who underwent abdomen-pelvis CT, no patient had a positive urine pregnancy test. Review of medical records after CT revealed that none of the women were pregnant at the time of CT scanning. Six (6/451) patients had a negative urine pregnancy test, 12 patients were either status post hysterectomy or tubal ligation (12/451), and 8 patients were post-menopausal (8/451). Written informed consent was obtained from 55 of 451 (12.2%) women of childbearing potential prior to their CT.

### 3.2 Clinical indications and justification

The top 5 clinical indications in our study included appendicitis (113/451, 25.1%), renal colic or calculi (69/451, 15.3%) obstructive uropathy (66/451, 14.6%), non-specific abdominal pain (37/451, 8.2%), and cancer staging (16/451, 3.5%). Although all CT examinations were deemed as indicated per the ACR Appropriateness Criteria, 315 of 673 (47%) phases were unjustified amongst the multiphase abdomen-pelvis CT examinations. The distribution of unjustified phases for multiphase CT was: non-contrast (122/673, 18%), arterial (33/673, 5%), portal venous (125/673, 19%) and delayed (35/673, 5%) phases. About 1.1 unjustified CT phases were acquired per patient amongst those who underwent multiphase CT. The most frequent clinical indications for unjustified portal venous phase included obstructive uropathy and renal colic or calculi. Appendicitis was the most frequent clinical indication for the acquisition of unjustified non-contrast phase.

### 3.3 Frequency of multiphase and single-phase CT in WOCBP

Nineteen patients (19/451, 4.2%) who underwent multiphase abdomen-pelvis CT were < 18-year-old (ages between 14 and 17 years). Of these, most patients (10/19 patients) underwent 2-phase abdomen-pelvis CT examinations and one patient (1/19) had a 3-phase abdomen-pelvis CT.

**Figure 1:**
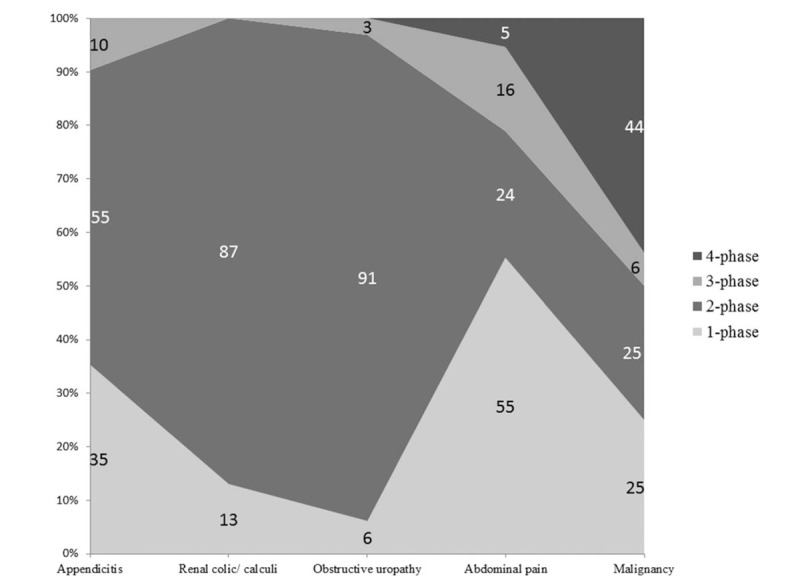
Top 5 most common clinical indications for abdomen-pelvis CT and the number of scan phases performed per patient.

Amongst the 451 women of childbearing potential who underwent abdomen-pelvis CT, multiphase CT was performed in 296 women (296/451, 65.6%) and the remaining had a single-phase non-contrast CT (155/451, 34.4%). Two-, 3-, and 4-phase abdomen-pelvis CT examinations were performed in 240 (240/451, 53.2%), 31 (31/451, 6.9%), and 25 (25/451, 5.5%) patients, respectively. Most 2-phase abdomen-pelvis CT examinations were acquired in non-contrast and portal venous phases. Figure 1 summarizes the distribution of the number of phases acquired for top five clinical indications.

### 3.4 Doses for multiphase CT

CTDIvol and DLP for single phase and multiphase abdomen-pelvis CT protocols are summarized in Table 1. Portal venous phase was associated with significantly higher radiation doses (CTDIvol and DLP) as compared to the other phases (*P* < .0001). Median total DLP for justified and unjustified phases were 266 (inter-quartile range 215) and 758 (inter-quartile range 539) mGy.cm, respectively. Unjustified phases were associated with a 65% higher radiation dose for patients who underwent multiphase abdomen-pelvis CT examinations as compared to those who had justified abdomen-pelvis CT phases (*P* < .0001).

**Table 1:**
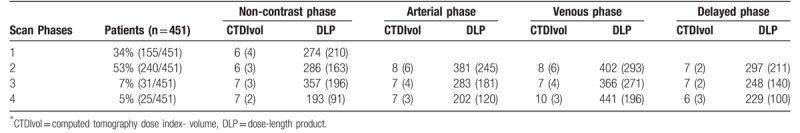
Median (and interquartile range) radiation doses (CTDIvol in mGy and DLP in mGy.cm) for single and multiphase abdomen-pelvis CT protocols. Portal venous phase CT was associated with significantly higher doses compared to the other phases (*P* < .0001). ANOVA for the DLP (*P* < .0001) and CTDIvol (*P* = .007) showed statistically significant difference.

## 4 Discussion

Almost two-thirds (296/451 patients) of women of childbearing potential who underwent abdomen-pelvis CT in our study had multiphase CT; close to one-half (47%) of phases were unindicated based on the ACR Appropriateness Criteria. Giannitto et al evaluated multiphase CT in 76 women of reproductive age and found that 47% of scan phases (93/197) were unindicated according to the ACR Appropriateness Criteria.^[10]^ While we found a similar distribution of unjustified non-contrast and portal venous phases (18%–19%), Giannitto et al reported a much higher proportion of unindicated non-contrast phase (45%) followed by the delayed and arterial phases.^[10]^ This discrepancy may be related to the higher prevalence of patients with renal colic/calculi and obstructive uropathy in our institution. Apart from non-contrast imaging, these patients were also scanned in the portal venous phase of contrast enhancement. In our study, unjustified portal venous phase was performed most commonly for obstructive uropathy and renal colic or calculi while unjustified non-contrast phase was performed for appendicitis. Although we do not know the reason for acquiring post-contrast CT in patients with such clinical indications, they may have stemmed from the unreliable clinical indications and a lack of proper supervision of CT protocols. Alternatively, the unjustified use of multiple phases may be due to the use of single abdomen CT protocol for different clinical indications. To mitigate use of unjustified scan phases, we recommend that the radiologists screen the clinical indications and specify what scan phases are indicated. The CT technologists must create and archive clinical indication or scan phase-based CT protocols on the scanners so that inadvertent errors or overuse of multiphase CT can be avoided. Despite these differences, our study had a similar proportion of unindicated phases (1.1 scan phases/patient) as compared to Giannitto et al (1.2 unindicated scan phases/patient).^[10]^

Guite et al also evaluated 500 abdomen-pelvis CT examinations and reported that about 53% (264/500) CT examinations had one or more unnecessary phases according to the ACR criteria.^[11]^ They also found that the most common unindicated phase (78%) was delayed imaging followed by non-contrast (12%) and arterial phase (11%) imaging. In our study, very few patients (5%) had delayed imaging. Guite et al reported an increase of 46% in radiation dose with the acquisition of unindicated phases compared to 65% in our study. Smith-Bindman et al have also reported a doubling of radiation doses for abdomen-pelvis CT with the acquisition of multiphase CT.^[11]^ Higher radiation dose penalty with unindicated phases in our study was likely related to the more frequent acquisition of unindicated portal venous phase imaging which is associated with a higher radiation dose compared to the unindicated non-contrast phase in Guite et al study.^[11]^ Frequent acquisition of multiphase abdomen-pelvis CT in pediatric patients (<18 years) in our study has also been reported in other studies.^[12]^ Rostad et al reported that more than half the abdomen-pelvis CT in children from 104 institutions had 2 or more scan phases.^[12]^ While the acquisition of multiphase abdomen-pelvis CT is frequent, prior studies have reported that additional phases do not increase the diagnostic information in most acute or chronic ailments.^[5–7,10,11]^

There are implications for our study. Although not pregnant, due to conservative social norms in Qatar, most single women of childbearing potential were requested to sign a written informed consent form prior to their CT. Following our study, for all women of childbearing potential undergoing CT, a pre-examination pregnancy determination form was implemented to query the women about the first day of their last menstrual period, and about the likelihood of pregnancy. Informed consent is now acquired from women who are pregnant and undergo CT scanning. Another implication of our study pertains to overuse of multiphase abdomen-pelvis CT in young patients despite guidelines on this subject.^[9]^ Unfortunately, most multiphase CT examinations were performed in patients with benign clinical indications that should be imaged with a single non-contrast phase CT, for example, in patients with flank pain or obstructive uropathy. Conversely, most contrast-enhanced abdomen-pelvis CT was performed after unindicated non-contrast phase through the entire abdomen and pelvis. A purported cause for frequent multiphase CT is unreliable clinical indications from the referring physician. While this may justify acquisition of some multiphase CT examinations, this reason does not apply in most patients with either routine or emergent clinical indications for abdomen-pelvis CT can be imaged with either non-contrast CT (for renal colic, obstructive uropathy or flank pain) or single post-contrast phase (unspecified abdominal pain, diverticulosis, or appendicitis).

The study highlights the need for raising awareness amongst both referring physicians and the radiologists about the need for correct clinical indications for abdomen-pelvis CT in the vulnerable and young patients included in our study. Although automatic tube potential selection (where available) and automatic tube current modulation techniques (on all scanners) were used in most patients, due to constant reference parameters, no significant change in radiation doses was noted between non-contrast, arterial or portal venous phases. A lack of a substantial difference between radiation dose for non-contrast phase for kidney stone versus for other clinical indications suggest that similar scan parameters and scan lengths are applied for both phases. Given the limited information content of non-contrast abdomen-pelvis CT, its radiation dose should be decreased in patients with suspected renal colic and its frequency should be reduced in patients undergoing post-contrast abdomen-pelvis CT.

Our study has limitations. This was a retrospective, observational study, and there were no interventions over this study. We limited our study to abdomen-pelvis CT due to larger variations and anticipated effects of associated radiation dose in women of childbearing potential from frequent multiphase CT protocols. The effect of different CT scanners on the associated radiation doses was not analyzed due to the limited sample size of matched clinical indications and patient sizes on different CT systems. We also believe that the effect of multiphase abdomen-pelvis CT which leads to 2 to 4 folds higher doses compared to single-phase CT likely offsets incremental differences in radiation doses from different scanners included in our study. Another limitation of our study pertains to a lack of local or national appropriateness or justification guidelines in Qatar which forced us to use the ACR Appropriateness Criteria to assess justification. This may have led to an overestimation of indicated CT exams and phases compared to unindicated examination. For example, ultrasound is generally the first line investigation in patients with renal colic whereas non-contrast CT is considered an appropriate test according to the ACR criteria.

In conclusion, multiphase abdomen-pelvis CT examinations were frequent but were rarely useful or indicated in our patients. Unfortunately, there were little differences between applied scan parameters and associated radiation doses between different scan phases when patients undergo multiphase abdomen-pelvis CT. To accomplish appropriate CT dose utilization, both the scan phases and scan parameters need a substantial change.

## Author contributions

**Conceptualization:** Mannudeep K Kalra.

**Data curation:** Huda Al Naemi, Antar Aly, Mohamad Hassan Kharita, Shatha Al Hilli, Amal Alobadli, Ramandeep Singh, Mannudeep K Kalra.

**Formal analysis:** Antar Aly, Ramandeep Singh, Mannudeep K Kalra.

**Funding acquisition:** Huda Al Naemi, Madan M Rehani.

**Investigation:** Huda Al Naemi, Mohamad Hassan Kharita, Shatha Al Hilli, Amal Alobadli, Ramandeep Singh, Mannudeep K Kalra.

**Methodology:** Huda Al Naemi, Mohamad Hassan Kharita, Shatha Al Hilli, Ramandeep Singh, Madan M Rehani, Mannudeep K Kalra.

**Project administration:** Huda Al Naemi, Antar Aly, Mannudeep K Kalra.

**Resources:** Antar Aly, Shatha Al Hilli, Amal Al Obadli.

**Supervision:** Huda Al Naemi, Madan M Rehani, Mannudeep K Kalra.

**Validation:** Antar Aly, Ramandeep Singh, Madan M Rehani, Mannudeep K Kalra.

**Visualization:** Huda Al Naemi, Mannudeep K Kalra.

**Writing – original draft:** Antar Aly, Ramandeep Singh, Mannudeep K Kalra.

**Writing – review & editing:** Antar Aly, Ramandeep Singh, Madan M Rehani, Mannudeep K Kalra.
